# The Potential Role of Cell Penetrating Peptides in the Intracellular Delivery of Proteins for Therapy of Erythroid Related Disorders

**DOI:** 10.3390/ph6010032

**Published:** 2013-01-07

**Authors:** Lefkothea C. Papadopoulou, Asterios S. Tsiftsoglou

**Affiliations:** Laboratory of Pharmacology, Department of Pharmaceutical Sciences, Aristotle University of Thessaloniki, Thessaloniki GR54124 Macedonia, Greece; E-Mail: tsif@pharm.auth.gr

**Keywords:** protein transduction, CPPs, erythroid related disorders, protein therapy

## Abstract

The erythroid related disorders (ERDs) represent a large group of hematological diseases, which in most cases are attributed either to the deficiency or malfunction of biosynthetic enzymes or oxygen transport proteins. Current treatments for these disorders include histo-compatible erythrocyte transfusions or allogeneic hematopoietic stem cell (HSC) transplantation. Gene therapy delivered via suitable viral vectors or genetically modified HSCs have been under way. Protein Transduction Domain (PTD) technology has allowed the production and intracellular delivery of recombinant therapeutic proteins, bearing Cell Penetrating Peptides (CPPs), into a variety of mammalian cells. Remarkable progress in the field of protein transduction leads to the development of novel protein therapeutics (CPP-mediated PTs) for the treatment of monogenetic and/or metabolic disorders. The “concept” developed in this paper is the intracellular protein delivery made possible via the PTD technology as a novel therapeutic intervention for treatment of ERDs. This can be achieved via four stages including: (i) the production of genetically engineered human CPP-mediated PT of interest, since the corresponding native protein either is missing or is mutated in the erythroid progenitor cell (ErPCs) or mature erythrocytes of patients; (ii) isolation of target cells from the peripheral blood of the selected patients; (iii) *ex vivo* transduction of cells with the CPP-mediated PT of interest; and (iv) re-administration of the successfully transduced cells back into the same patients.

## 1. Introduction

Erythropoiesis is a complex developmental process occurring in bone marrow (BM) during adult life. Pluripotent hematopoietic stem cells (HSCs) upon differentiation give rise to erythroid progenitor cell (ErPCs) (including eythroid committed burst-forming units-erythroid ‒ BFU-Es and colony-forming units-erythroid ‒ CFU-Es), which are eventually differentiated into mature erythrocytes or red blood cells (RBCs). Erythropoiesis involves: (i) activation of the erythropoietin (EPO) receptor mediated signaling by EPO; EPO is promoted by hypoxia via the hypoxia-inducible transcription factors; (ii) activation of heme biosynthesis; (iii) expression of globin genes; and (iv) completion of terminal morphological maturation [1,2]. Under normal erythropoiesis, hemoglobin A (HbA: α_2_β_2_) and, to a much lesser extent, hemoglobin A_2_ (HbA_2_: α_2_δ_2_) as well as fetal hemoglobin (HbF: α_2_γ_2_) are produced in human adult RBCs. Each one of the globin subunits [alpha (α), beta (β), delta (δ) or gamma (γ)] carries one molecule of heme (an iron protoporphyrin IX) as a prosthetic group, able to bind and transport the oxygen into tissues [3,4].

Human mature erythrocytes lack nucleus and are among the most abundant human cell types (~2-3 × 10^13^). They have a biconcave shape, which offer them flexibility during blood circulation and make them able to penetrate through basement membranes during their transit from the splenic cord to splenic sinus and through the microvasculature bed during their life span in circulation (100 - 120 days) [5,6]. Normal RBCs are indeed quite tolerant to hemolysis.

Normally, ~280 × 10^6^ molecules of adult haemoglobin (HbA) are generated in ErPCs [7]. Alpha globin chain is co-translationally regulated with heme [8,9]. Heme-containing alpha globin chain associates with apo-beta globin chain and drives the insertion of heme into it [10].

Erythroid related disorders (ERDs) in most cases result either from reduced RBC production due to ineffective erythropoiesis and failure of maturation of the ErPCs (like in sideroblastic anemias, thalassemias) or increased RBC destruction, associated with hemoglobinopathies as well as enzyme deficiencies (like those occurring in porphyrias) [3,4,11,12]. There are also other disorders that affect mainly hematopoietic progenitor cells, like the myeloproliferative polycythemia vera, the acute erythroid leukemia and the myelodysplastic syndromes. The latter are characterized by overwhelming or ineffective erythropoiesis [13], accompanied by known and unknown genetic abnormalities. Fanconi anemia (FA) is another genetic disorder associated with mutations in at least twelve genes that could lead to aplastic anemia [14]. Unfortunately, there is no current standard therapy of all ERDs. For example, there is no cure for sideroblastic anemias, while symptomatic therapy is applied in patients with porphyrias. Hemoglobinopathies, currently, are treated symptomatically by long-life erythrocyte transfusions.Cure of ERDs could be achieved by: (a) correction of the abnormal gene or replacement with a normal one; and (b) transplantation with allogeneic HSCs from HLA - matched donors or with autologous HSCs, gene-corrected previously *ex vivo* [15,16]. Unfortunately, the poor availability of transplants from HLA - matched donors, the severity of graft *versus* host disease (GVHD) that can be developed in cases of allogeneic HSC transplantation (HSCT) and the complexities associated with gene therapy (like insertional mutagenesis - tumorigenesis) demand alternative therapeutic interventions for ERDs.

Enzyme or protein replacement therapy (ERT/PRT) via the Protein Transduction Domain (PTD) technology could be considered as an alternative intervention that, compared to gene therapy, is regarded safe, since it doesn’t affect the host structural integrity of the genome [17,18,19]. PTD technology is based on the ability of short aminoacid (aa) sequences [CPPs or PTDs] to facilitate intracellular delivery of recombinant proteins (as well as other molecules) conjugated-linked to them. This remarkable technology permits the development of a novel class of proteins therapeutics (CPP-mediated PTs) for certain genetic and/or metabolic disorders, including ERDs that are discussed later on.

In the framework of this “concept paper”, we wish: (i) to discuss the pathophysiology and current treatment - management of some ERDs (sideroblastic anemias, porphyrias and hemoglobinopathies); and (ii) to present how CPP-mediated PTs (biodrugs) can be used as an alternative ERT/PRT approach for treating ERDs.

## 2. Genetic and Metabolic ERDs and current Treatment

### 2.1. Sideroblastic Anemias

Under normal erythropoiesis, erythroblasts contain ~200 mitochondria per cell, playing crucial role in iron metabolism and biosynthesis of heme and iron-sulphur [Fe-S] clusters [20]. More than 80 percent of body heme levels are synthesized in ErPCs (BFU-Es, CFU-Es) for the production of hemoglobin [21], which corresponds to ~ 40-50% of total protein in mature RBCs. Disruption of interconnection between heme biosynthesis, [Fe-S] biogenesis and iron - heme proteins could result in congenital anemias (CGAs) [20].

Sideroblastic anemias are either acquired (associated with drugs or alcohol abuse) or congenital. They are characterized by the appearance of ringed sideroblasts (erythroblasts characterized by the accumulation of non-heme iron in perinuclear mitochondria) as common feature in the BM [20,22].

Mutations in the erythroid-specific heme biosynthetic gene *ALAS-2* (δ-aminolevulinic acid synthase - 2, isoform exclusively synthesized in ErPCs) can lead to X-linked sideroblastic anemia (XLSA) (OMIM: 300751), the most common CGA. More specifically, some mutations intervene with the binding of ALAS-2 to the succinyl-CoA synthetase that provides the succinyl-CoA, necessary for the synthesis of δ-aminolevulinic acid (ALA), the first enzyme in the biosynthesis of heme [23,24]. Patients carrying mutations in *ALAS-2* that influence the affinity of the enzyme for its cofactor pyridoxal phosphate may respond to treatment with pyridoxine (vitamin B6) [25,26,27].

Sideroblastic anemias may also occur by mutations in genes encoding transporters, like: (a) the solute carrier SLC25A38, an erythroid specific transporter of glycine into the mitochondrial matrix (OMIM: 205950) [28,29], where with succinyl-CoA will give genesis to ALA; and (b) the inner mitochondrial membrane ABCB7 transporter [30]. This transporter controls the availability of mitochondrial iron to be inserted in the protoporphyrin IX via ferrochelatase (FECH) [25,29]. Mutations in ABCB7 transporter may disrupt the maturation of [Fe-S] [31] in FECH during heme biosynthesis and lead to X-linked sideroblastic anemia with ataxia (XLSA-A) (OMIM: 301310). There is also evidence that deficiency of glutaredoxin 5 (GLRX5), also essential for [Fe-S] biogenesis, can lead to sideroblastic anemia in humans (OMIM: 609588) [20,32].

Furthermore, congenital sideroblastic anemias are associated with abnormalities concerning mitochondrial protein synthesis, like: (a) Pearson Marrow-Pancreas Syndrome (PMPS) [33] (OMIM: 557000), with half patients being heteroplasmic for the 4977-bp common deletion in the mitochondrial genome; (b) Mitochondrial myopathy with Lactic Acidosis and ringed Sideroblasts (MLASA) [34] (OMIM: 600462), with mutations in *PUS1* gene, encoding PUS playing role in the metabolism of uridine; and (c) Thiamine Responsive Megaloblastic Anemia (TRMA) [35] (OMIM: 249270) due to mutations in thiamine transporter SLC19A2. Unfortunately, there is no cure for congenital sideroblastic anemias up to now, while gene therapy is considered as a possible future therapeutic intervention [20].

### 2.2. Porphyrias

“Porphyrias” represent a group of inherited or acquired disorders attributed to deficiencies of enzymes involved in heme biosynthesis. Porphyrin intermediates (substrates for the corresponding defective heme biosynthetic enzymes) accumulate in the BM erythropoietic cells and/or in the liver. Thus porphyrias are classified as erythropoietic or hepatic, respectively. Patients with porphyrias must avoid the use of the so called “porphyrogenic” drugs, which may enhance the frequency of acute porphyria attacks with serious complications [36,37].

More specifically, mutations in human heme biosynthetic genes can give genesis to porphyrias [38], like in: (i) *ALAS-2,* leading to X-linked dominant erythropoietic protoporphyria (XLEPP or XLDPP) (OMIM: 300752) [39,40]; (ii) *UROS* (uroporphyrinogen III synthase), leading to congenital erythropoietic porphyria (CEP) (OMIM: 263700) and (iii) *FECH*, leading to erythropoietic protoporphyria (EPP) (OMIM: 612386). Moreover, mutations in human *GATA-1* can also result in CEP (OMIM: 305371) [41]. It is worth mentioning here that at least five heme biosynthetic enzymes have binding site for the GATA-1 (an erythroid specific transcription factor) [42].

Acute porphyria attacks are life threatening and can be manipulated by: a) avoiding precipitating factors [porhyrinogenic drugs (chloroquine, sulphonamide antibiotics, rifampicin), alcohol, hormones (estrogen, progesterone) that overproduce porphyrins)]; b) alleviating the neurological (nausea, vomiting, pain) or cardiovascular problems with symptomatic therapy; c) intravenous (i.v.) administration of hemin, in the form of heme arginate or hematin [38]. However, treatment with hemin should be monitored, since free heme accumulation could lead to iron overload and organ failure. Patients with chronic porphyrias (like EPP, CEP) present mild to severe cutaneous lesions (erythema, blistering and others) that exacerbated after expose to sunlight. Thus, in chronic porphyrias, intentional avoidance of sunlight is recommended. Liver or BM/HSC transplantations are recommended in severe cases [36,38,43].

### 2.3. Hemoglobinopathies

Hemoglobinopathies [44], as inherited disorders of hemoglobin, are among the most common monogenetic ERDs. Hemoglobinopathies reflect the natural selection of carriers, being protected from the consequences of infection with *P. falciparum* that causes malaria [45,46,47,48]. A plethora of hemoglobinopathies, listed in the HbVardatabase [49], arise from globin gene mutations. About 5-7% of the world’s population carries globin gene variants, while ~340.000 annual births appear with major hemoglobin disorders [7,47,50].

#### 2.3.1. Thalassemias

Among the hemoglobinopathies, thalassemias [4] are a group of disorders named from the Greek word *“thalassa”* for sea, since they have been endemic in the areas around Mediterannean Sea [51]. In thalassemias, absence or reduced amount of one or more globin chains [necessary for the formation of the tetramer α_2_β_2_ of HbA] lead to decreased HbA, hypochromia and microcytosis. Imbalance between alpha and beta globin chains, leads to ineffective erythropoiesis in BM, hemolysis in peripheral RBCs and thus mild or severe hemolytic anemia [50]. In alpha thalassemias [52] (OMIM: 141750), partial (α^+^) or total (α°) deletions of one or more of the four alpha globin genes are the most common causes, while beta thalassemias (OMIM: 613985) are attributed to more than 200 mutations (point mutations, insertions or inversions-deletions) associated either with some beta globin synthesis (β^+^) or no synthesis at all (β°) [47].

In alpha thalassemia, unpaired beta and gamma globin chains usually don’t precipitate but rather polymerize to form unstable tetramers, β_4_ in HbH or γ_4_ in HbBart’s. HbBart’s results in fatal hydrops fetalis [46,51]. Both HbH and HbBart’s have very high affinity for oxygen and cannot support transport of oxygen to tissues. Moreover, HbH and HbBart’s have been found to cause increased apoptosis and reduced cell survival of ErPCs in BM. These apoptotic cells are removed via phagocytosis [46,50].

In case of beta thalassemia, defects in both genes lead to either intermedia beta thalassemia or beta thalassemia major or, otherwise, β°-thalassemia or Cooley’s anemia. Severity of syndrome increases as the beta globin chain production is reduced, while alpha globin chain production is at normal levels. This discrepancy leads to excess amounts of free alpha globin chains in both the ErPCs in BM and the RBCs in the peripheral blood. Free alpha globin chains finally precipitate, form inclusion bodies, destruct the membranes and lead to cell death of RBCs (promoted by iron and free heme), ineffective erythropoiesis and anemia [46].

Patients with beta thalassemia major (hemoglobin levels lower than 70 g/L) need long-life, safe, immunocompatible (to avoid alloimmunization), fresh units of erythrocyte (leucocyte reduced) transfusions every 2^nd^ to 4^th^ week to correct anemia. In patients with intermedia beta thalassemia, transfusions depend on the severity of situation and, usually, is episodic. Regular erythrocyte transfusions usually result in iron overload, which could be toxic due to accumulation in various organs (liver, myocardium) and can lead even to cardiac insufficiency [50]. Thus, chelation therapy (e.g. parenterally administered desferrioxamine or new oral chelating agents, like deferriprone) is absolutely needed to remove excess iron from the human body [46].

Αnother therapeutic approach is the epigenetic modulation of globin gene expression and induction of HbF to therapeutic levels with the use of EPO, HU as well as other pharmacological agents. EPO therapy stimulates erythroid proliferation, decreases apoptosis of erythropoietic cells and finally increases hemoglobin levels by ~25 g/L [53]. Drugs, like hydroxyurea (HU, hydroxycarbamide), sodium butyrate or aza-cytidine, can activate Gγ-globin and enhance fetal HbF production, thus being quite useful [46,54]. HU has been approved by FDA to stimulate HbF synthesis. HU is an antimetabolite that transient arrest DNA synthesis in the late ErPCs, thus giving advantage to premature earlier ErPCs, which have active the HbF program [55,56]. It is known, that human gamma globin genes are hypomethylated in fetal tissue and thus are transcribed, while transition to adult life results in their methylation and subsequent silencing [57]. Short chain fatty acid derivatives, like butyrates, inhibit histone deacetylases, increase acetylation of core histones, alter the chromatin structure and permit accession of the transcription factors in the promoters of gamma globin genes. Increased transcription levels lead to increased HbF levels.

HSCT can be applied mainly in low risk patients (based on age, gender, physical situation, other pathological conditions) as curative therapy [58]. The main problem, however, is the availability of transplants from HLA - matched donors, since allogeneic HSCT itself can lead to GVHD as well as others serious complications. The use of umbilical cord blood transplants would extend the donors pool [59].

Gene therapy could be used instead, but this approach has its own limitations, since it has to be safe by avoiding insertional mutagenesis and leukemogenesis, to be specific in erythropoietic cells and to achieve adequate production of beta globin. Cell and gene therapy in patient-specific embryonic stem cells (ESCs) or induced pluripotent stem cells (iPSCs) by correcting the mutant gene and subsequent transplantation into the patient could overcome a number of technical obstacles [60]. Thus far, only Leboulch’s team succeeded in a gene therapy approach (using a lentiviral βeta globin gene transfer) to achieve blood transfusion independence for four years now in a patient with severe beta thalassemia, being dependent since early childhood on monthly transfusions [54,61]. Moreover, intensive investigation is on progress to delineate where and how the beta globin gene was integrated and remained functional.

RNA interference technology could offer advantage in gene therapy approach by silencing the alpha globin gene to reduce the alpha globin levels and restore normal ratio between alpha and beta globin chains [62]. Correction of the mutant phenotype at the level of mRNA could be also approached, by using chemically stabilized antisense oligonucleotides [60,63].

#### 2.3.2. Sickle Cell Anemia

Sickle cell anemia (OMIM: 603903) or, otherwise, sickle cell disease (SCD), is a genetic disorder that results from a single point mutation (T to A) in the 17^th^ nucleotide of the coding sequence (CDS) of the beta globin gene, producing the abnormal hemoglobin S (HbS: α_2_β_2_^s^) [64]. The resulting replacement of the negatively charged glutamic acid (6^th^ aa) by valine, a non-polar neutral aminoacid, creates a hydrophobic motif, which under hypoxia conditions leads to binding between the beta chains of two deoxygenated HbS tetramers. This hemoglobin polymerization disrupts the architecture - flexibility of RBCs. The rigidity of RBCs leads to vaso-occlusion, hemolysis and chronic anemia. Elevating the HbF levels seems to offer good results in cases of SCD, since HbF and α_2_γβ^s^ tetramers are not involved in the polymerization process induced by HbS. Novel therapeutic candidates can induce gamma-globin gene expression *in vivo* [65]. RNA interference technology could be also applied to silence the beta^s^-globin gene and reduce the polymerization in hypoxic conditions [66]. Gene-corrected BM/HSC cells derived from mouse iPSCs were able to treat SCD upon transplantation in animals [67]. Furthermore, regenerative medicine would be considered as an alternative treatment for some of the ERDs by *in vitro* generation of mature, functional RBCs [68] or *ex vivo* expansion of normal RBCs [69]. Of course, there are limitations, either due to the huge amounts of RBCs, needed each time for transfusion, or due to the high cost of such an approach.

## 3. Protein Transduction Domain Technology for intracellular DELIVERY of Protein Therapeutics (PTs)

“Omics” technologies (like genomics, transcriptomics, proteomics, metabolomics, interactomics), implemented by bioinformatic analysis, contributed to the development of a large number of new therapeutic targets, the majority of which are located intracellularly.

Protein therapy as direct delivery of the gene translational product itself, the PT, could be considered as an alternative therapeutic approach to gene therapy of monogenetic or metabolic disorders [18], like in the case of insulin as PT in diabetes mellitus. However, in many cases, PTs have to “cross” the plasma membrane of the cells [70] and even to target a specific organelle (e.g. mitochondria) [18] in order to exert their therapeutic effect in diseases, where a mutated gene encodes either an abnormal intracellular protein or no protein at all.

PTD technology developed over the past decades is based on the intrinsic capacity of some native proteins to penetrate biological membranes. Proteins, like the TAT protein of the HIV-1 virus (a transactivator factor of transcription) [71] and the Antennapedia, a *Drosophila* homeotic transcription factor [72], were found to be able to penetrate the plasma membrane [73] and carry on heterologous proteins [71] intracellularly.

PTD technology uses small peptides (CPPs or PTDs) of less than 30 aa in length, able to penetrate almost all biological membranes and carry on intracellularly a variety of cargos ranging from small molecules (drugs, oligonucleotides, siRNAs) to macromolecules (proteins, plasmids, liposomes and nanoparticles) [74,75,76,77,78]. CPPs are either protein derived peptides (like the eleven amino acid TAT peptide from the HIV-1 TAT full length protein) or synthetic peptides (like the R9) or even chimeric peptides (like the transportan) (Table 1) [18,79,80]. A CPPsite has been uploaded, offering information (origin, sequence, subcellular localization) for more than 800 CPPs [79].

CPPs are in some cases cationic in nature (like the TAT peptide and the chemically synthesized polyarginines R7, R8, R9), in other cases amphipathic, bearing both cationic and hydrophobic residues (like the penetratin, VP22) or even hydrophobic (like the PFVYLI peptide), as also shown in Table 1.

**Table 1:** Representative CPPs.

CPP	Origin	Aminoacid (aa) Sequence /Physicochemical nature	Length(aa)	Refs
TAT	HIV-1 TAT (transactivator factor of transcription)	YGRKKRRQRRR Cationic peptide	11	[73,81]
Penetratin (Antp)	*Drosophila* homeotic transcription factor encoded by *antennapedia* gene	RQIKIWFQNRRMKWKK Amphipathic pepttide	16	[72]
VP22	herpes simplex virus VP22 transcription factor	DAATATRGRSAASRPTERPRAPARSASRPRRPVD Amphipathic peptide	35	[82]
poly- Arginines	chemically synthesized	R7, R8, R9 Cationic peptides	9 / 8	[83,84]
Transportan	galanin -mastoparan	GWTLNSAGYLLGK-INLKALAALAKKIL Chimeric - Amphipathic peptide	27	[85]
TP10	truncated form ofTransportan	AGYLLGKINLKALAALAKKIL Chimeric - Amphipathic peptide	21	[86]
pVEC	Vascular Endothelial (Ve) - Cadherin	LLIILRRRIRKQAHAHSK Amphipathic peptide	18	[87]
Pep-1	Trp-rich motif-SV40 NLS	KETWWETWWTEWSQPKKKRKV Chimeric - Amphipathic peptide	21	[88]
C105Y	peptide based on the residues 359-374 of alpha1-antitrypsin	CSIPPEVKFNKPFVYLI Amphipathic peptide	17	[89]
PFVYLI	derived from the synthetic peptide C105Y	PFVYLI Hydrophobic peptide	6	[89]
CADY	chemically synthesized, combining aromatic (W) and cationic (R) residues	GLWRALWRLLRSLWRLLWRA Amphipathic peptide	20	[90]
CAPHs	chemically synthesized with cationic amphiphilic polyproline helices	P11LRR to P14LRR Cationic - Amphipathic peptides	14 to 17	[91]

For cationic CPPs [enriched in arginines (R)], cellular entry is initiated by electrostatic interactions and hydrogen bonding between the arginine’s guanidine group and the cell surface’s negatively charged groups (like heparin sulphate proteoglycans, phosphates or carboxylates). Subsequently, endocytosis driven delivery model and, more specifically, macropinocytosis [92], seems to be the one most widely accepted model for cell penetration. Other models of endocytosis have also been proposed, depending not only on the nature of the used CPP, but also on the nature (sequence, length, conformation) of cargo as well as on the targeted cell type and the CPP to cell ratio employed [18,92,93,94,95]. Even extremely hydrophobic peptides, like the PFVYLI (the C-terminal sequence of the C105Y peptide) or the LLIIL (the N-terminal sequence of the pVEC CPP peptide) (Table 1), are important for the penetration of the cell membrane [89,96]. Aromatic residues, like tryptophans (W), as part of the hydrophobic domain of penetratin and CADY, are also proposed to play significant role in the transduction process.

Enhancement of intracellular delivery by 100x fold can be achieved by increasing the multivalency, which means coupling more than one CPP peptides (e.g. fifteen TAT peptides) to cargo [97]. Multivalency has also been found to increase the efficiency of binding to cell surface and thus enhance the intracellular uptake [19].

### 3.1. Production of PTs via PTD Technology

CPP-mediated PTs are engineered via PTD Technology by: a) cloning of the CDS, corresponding to the open reading frame of the PT of interest, adjacent to the sequence encoding the CPP (like the TAT peptide) into a suitable expression vector (like the pTAT/TAT-HA from Dowdy’s group); b) subsequently transformation of appropriate competent (bacterial) cells;and c) production of fusion proteins containing the CPP of choice linked to the protein of interest [81,98,99]. In cases that the produced CPP-mediated PT needs post-translational modifications in order to exert its biological actions, novel modified eukaryotic cloning-expression systems are employed [100], since bacteria fail to carry out post translational modifications (e.g. glycosylation) of the produced protein.

Chemical conjugation can be also applied between the CPPs and the cargo of interest in order to obtain the CPP - cargo, which will be able to deliver and, later on, release the cargo (e.g. the desired PT) intracellularly, depending on the environmental pH, temperature or protease [84,101,102,103,104]. The most widely used linker for conjugation is the disulphide bridge, permitting reduction of the CPP-cargo after penetration of the cell membrane, due to the high concentration of intracellular glutathione and subsequent release of the cargo. However, a recent study proposed that such a reduction could also happen at the level of cell surface [105]. Other linkers proposed for chemical conjugation of the PTs with the CPP of interest are the maleimide linker bond, sulphanyl (bi-functional) bond as well as phosphatydylethanolamine hydrogen bonding [95,106].

Protein transduction can be also approached in a non covalent manner by either premixing of the selected CPP with the desired cargo or by formulating the CPP - cargo in liposomes or nanoparticles [107,108,109]. Non covalent molecular interactions between the CPPs and the cargo seems to depend on their tertiary structures and hydrophobic as well as electrostatic interactions [107].

### 3.2. Pharmacokinetic, Toxicity and Targeting of CPP-Mediated PTs

PTD Technology, compared to gene therapy, is regarded safe, since it doesn’t affect/alter the host genome integrity [17,18,19], although obstacles remain to be solved. These obstacles include: (i) lack of tissue specificity; (ii) protein instability, induced by proteases being present in circulation and inside the cells; (iii) immunogenicity; and (iv) other adverse reactions (like hypersensitivity) of the delivered CPP-mediated PTs (Table 2).

**Table 2:** Obstacles of CPP-mediated PTs.

Obstacles of CPP-mediated PTs	Possible solution of the obstacles	Refs
lack of tissue specificity	Development of “smart” delivery platforms	[95]
	Use of target-specific antibodies, attached via bonds sensitive to cell environmental or external stimulus conditions	[95,104]
	Use of Homing peptides	[110,111]
	Development of tissue specific CPPs	[112]
protein instability	Exchange L-amino acids of CPPs with the corresponding D-amino acids	[113]
	Polymerization technologies (protein PEGylation)	[114]
	Construction of liposomes and nanoparticles	[95]
immunogenicity	de-immunization by substitution of key aminoacids of T- or B-cell epitopes on CPP-mediated PT, using computer algorithms	[115,116]
	Dosage / Route of administration (topical / i.v.)	[117]

Most of the CPPs applied for pharmacokinetic studies undergo rapid clearance from the blood, with liver and kidney considered as the primary organs of their accumulation [118]. The necessity for multiple times administration of CPP-mediated PTs at high doses may indeed increase their immunogenicity [117]. Stability of CPP-mediated PTs could be affected by exchanging the L-amino acids with the corresponding D-amino acids, although this procedure may reduce the intracellular delivery [113].

Polymerization technologies (e.g. protein PEGylation) as well as entrapment of proteins in liposomes and/or nanoparticles could achieve better pharmacokinetic profile for the CPP-mediated PTs and overcome a number of obstacles, allowing penetration of even intact biological barriers, like BBB or BSCB (blood-spinal cord barrier) [114].

The ability of CPPs to penetrate almost all biological membranes makes the targeted delivery of proteins a very important issue in order to minimize undesirable effects. One should take in consideration the special characteristics of the targeted organ microenvironment. Torchillin’s group [95,104] developed “smart” delivery platforms for organ-tissue specific targeting, after administration of the desired cargo (e.g. the PT of interest) in the circulation: CPP - cargo is shielded with a protective molecule, like PEG (polyethylene glycol polymer), thus enhancing solubility, decreasing accessibility for the circulating proteolytic enzymes and increasing the half time of the PT by reducing kidney filtration due to its increased size. PEG chains can carry target-specific antibodies, attached to the surface via bonds sensitive to cell environmental (e.g. pH, temperature, matrix metalloproteinases) or external (magnetic field, ultrasound) stimulus conditions. After accumulation of the “smart” nanocarrier in the targeted organ and release of the PEG-antibody conjugates, the exposed CPP permits the transduction of the cargo intracellularly. Such an approach seems reasonable for the design of tumor-specific drugs/prodrugs/biodrugs, although the observed cell heterogeneity in tumors raises a number of limitations.

Another interesting approach for targeting delivery could be the ability to attach, during the production process, a homing peptide, if any available, adjacent to the sequence of the suitable CPP-mediated PT of interest [110]. Such homing peptides, able to bind specific molecules on the targeted cell population and facilitate selective transduction of PT, could be identified by Phage Display technology [111]. The design of a CPP, specific for glial cells, has been presented as a promising therapeutic approach for glial-related disorders [112].

In cases of subcellular organelle-targeted therapy, specific targeted sequences [like NLS (nuclear localization signal) or MTS (mitochondrial targeting signal)]) can be inserted in the designed sequence (e.g. CPP-MTS-PT) [18,99,119,120,121]. In an *in vivo* mouse (E3-deficient) model, intramitochondrial delivery of lipoamide dehydrogenase (LAD; E3), fused to TAT peptide, restored LAD activity [122]. Another class of CPPs, the cationic polyproline helices (CAPHs), has been also proposed to reach even more efficiently the mitochondria via direct transport mechanism [91].

CPPs have been administered by various ways, both parentally as well as topically. Parental administration is the most frequent route for the delivery of biodrugs [107,110]. Parental administration is the most frequent route for the delivery of biodrugs. Oral delivery would open new opportunities in biodrugs’ administration, since it is non invasive and friendly to the patient for self administration, but it is not as yet applicable. The lack of specificity of CPP-mediated therapeutics towards various tissues till now led topical applications to be more preferable for clinical purposes [118]. Thus, CPP-mediated therapeutics (like PsorBan, RT-001, AZX-100), that have reached clinical trials, are mainly for local use towards scarring, psoriasis, pain, wrinkles and excessive sweating (see Table 3) [74,96,123,124]. Data from Phases I/II on more than 2,000 patients indicated no significant adverse reactions [125]. There are also clinical trials targeting heart problems or even cancer (Table 3). The so-called “smart” delivery platforms, bypassing stability and tissue specificity problems, would further facilitate the development of novel therapeutic interventions [95].

### 3.3. Targeted Intracellular Delivery of CPP-Mediated PTs in ErPCs and RBCs:Future ex vivo Therapy

It has been quite clear that ERDs, described thus far, include diseases, where ErPCs or even mature RBCs are deficient in a gene or its translation product and thus fail to function properly. Therefore, it is reasonable to assume that ERT/PRT approach would be beneficial for these disorders. An enzyme or any other type of missing protein can be produced as recombinant protein outside the body in a selected gene cloning-expression system, modified accordingly via PTD technology to carry an appropriate CPP and then be transduced into ErPCs or RBCs (wherever is needed) to deliver its intended function.

**Table 3:** The status of some Preclinical and Clinical trials of CPP-mediated therapeutics.

Biopharmaceutical Company	Product	CPP-cargo	Indication	Clinical Phase	Refs
CellGate, Inc.	PsorBan	R7-cyclosporin A	topical treatment of psoriasis	II (discontinued)	[126]
Revance Therapeutics, Inc.	RT-001	TAT-botulinum toxin (TransMTS TM platform technology)	topical treatment of wrinkles and excessive sweating	IIb I	[95,124]
Capstone Therapeutics	AZX-100	YARAAARQARA - HSP20 phosphopeptide	excessive dermal/keloid scarring and fibrotic disorders	II	[127]
KAI Pharmaceuticals	KIA-9803 KIA-1678 KIA-1455	TAT-protein kinase Cδ inhibitor TAT-protein kinase Cε inhibitor TAT-protein kinase Cε activator	myocardial infarction pain cytoprotection ischemia	I/II	[95,124]
Avi Biopharma	AVI-5038	(R-Ahx^1^-R)4 AhxB –PMO^2^	Duchenne Muscular Dystrophy/viral infections	Pre-clinical studies in mouse	[128]
Centro Nazionale AIDS-Istituto Superiore di Sanità -NovartisVaccines	Trial ISS P-002	TAT- V2-deleted Env proteins	HIV Infection	I	[129]
Traversa Therapeutics and Sanofi-Aventis	PTD–DRBD	PTD-DRBD^3^- siRNA	RNAi delivery	Pre-clinical studies	[130,131]
Xigen Pharm (Epalinges, Swiss)	XG-102 (D-JNKI-1)	TAT- JNK-inhibiting peptide	inflammatory bowel disease	Pre-clinical studies	[132]
Diatos – Drais Pharmaceuticals	DTS-108	DPV1047^4^- SN38 (activemetabolite ofirinotecan)	cancer treatment	I	[133]

^1^ Ahx:6-aminohexanoic acid, ^2^ PMO: morpholinooligomer, ^3^ DRBD: double-stranded RNA binding domain, ^4^ DPV1047: DPV (for Diatos Peptide Vectors) Vectocell peptide :CVKRGLKLRHVRPRVTRMDV (20aa)

This task, of course, requires careful study of each particular case of ERDs in order to design the appropriate experimental approach. A number of questions to be considered include: (i) what is the pathobiology of a given ERD; (ii) what type of ErPCs are involved; (iii) how are they characterized in terms of their cell surface antigens and progeny; (iv) what is the missing protein and its corresponding gene; (v) where is this protein located inside the cells; (vi) how does this protein function; and (vii) how shall this protein (PT) of interest be delivered and remain functional (in the form of a newly produced recombinant CPP-mediated PT) for relatively long time.This valuable information is considered essential prior to any design of experiments in order to clone, express and produce the CPP-mediated PT of interest in a highly purified state, biologically active and able to exert its function upon delivery into the target cells. A critical step for the successful treatment of ERDs by protein transduction is the isolation of the candidate recipient cells (target cells), deficient in the protein of interest, prior to transduction experiments. Current technologies, including flow cytometry and cell sorting complemented by functional assays, will be able to facilitate isolation of these target cells. These cells can be removed from the peripheral blood of patients, mobilizated by G-CSF, as it has been followed for HSC-based gene therapy for beta thalassemia [134]. Following characterization, isolated cells will be grown in culture at selective media with minimal manipulation and then be efficiently transduced with the CPP-mediated PT of interest for as long as needed. By the end of loading period, cells will be assessed for the efficiency of protein transduction and functionality, using conventional assays. Stability and biological activity of the transduced CPP-mediated PT are essential to correct the corresponding cellular protein deficiency and allow cells to act properly. Whether the transduced CPP-mediated PT will survive for as long as needed to correct the deficiency in the recipient cells is an issue for investigation. It may be possible for such cells to be submitted to repeated protein transductions in timed intervals. For example, ErPCs and/or RBCs, removed from patients, could be transduced *ex vivo* with the CPP-mediated PT of interest. It has to be tested whether transduction will affect the structural integrity and function of ErPCs and/or RBCs, causing hemolysis or other adverse effects. Furthermore, the transduced CPP-mediated PT is expected to enter the intracellular space and interact properly with other proteins or form functional protein complexes. Full comparability studies using deficient and transduced erythropoietic cells will be carried out in parallel to confirm that protein transduction delivers the intended therapeutic action. Successfully transduced, with the CPP-mediated PT, cells will then be administered back into the patients. In ERDs with aberrant protein located in the mitochondria, like in cases of mitochondrial enzymes ALAS-2 and FECH in X-linked sideroblastic anemia, X-lnked erythropoietic porphyria or erythropoietic protoporphyria, intramitochondrial targeted protein therapy via PTD technology should be applied [18,119].

Possible therapy of ERDs with the use of CPP-mediated PTs is still in its conceptual stage. At present, efforts can be made: (a) to produce erythroid related proteins (e.g. enzymes or oxygen carrier proteins) as fusion recombinant proteins bearing a CPP; and (b) to transduce them into either HSCs and/or ErPCs - RBCs. Basic studies on protein intracellular delivery, followed by preclinical studies with the use of relevant animal models, are needed to be evaluated, before clinical trials aimed to treat ERDs via CPP-mediated PTs would follow in the future. It is interesting, however, to underline that the field of CPP-mediated therapeutics (biodrugs) keeps growing in other areas of pathological disorders. Table 3 illustrates animal and human studies carried out or being on progress to assess the impact of the PTD technology in the treatment of such disorders. Of course, the roadmap of the development and application of PTD technology “from bench to bed” in people with ERDs is quite long, but is provocative and can be eventually fruitful.

## 4. Conclusions and Perspectives

The development of PTD technology, that uses small peptides (known as CPPs or PTDs) to enable protein cellular uptake into mammalian cells, opened new horizons in ERT/PRT approach of genetic/metabolic diseases. ERDs, like porphyrias, congenital sideroblastic anemias and hemoglobinopathies, result from deficiency of heme biosynthetic enzymes, impaired iron metabolism and absence or mutated globin chains, respectively. There is no current standard therapy of ERDs. However, CPP-mediated PRT emerges as an alternative therapeutic intervention for these disorders, which should follow all the way from “the bench to the bedside”. The results from the ongoing preclinical and clinical trials with CPP-mediated therapeutics in a variety of other disorders can be useful in order to predict the potential of outcome of CPP-mediated PTs in the treatment of ERDs.

## Abbreviations

aa: aminoacid
ALAS: ALA Synthase
BFU-Es: burst-forming units-erythroid
BM: bone marrow
CDS: coding sequence
CEP: congenital erythropoietic porphyria
CFU-Es: colony-forming units-erythroid
CGAs: congenital anemias
CPP: Cell Penetrating Peptide
CPP-mediated PTs: PTs engineered via PTD Technology
ErPCs: Erythroid progenitor cells
ERDs: Erythroid related disorders
ERT: enzyme replacement therapy
EPO: erythropoietin
EPP: erythropoietic protoporphyria
FA: Fanconi anemia
FECH: Ferrochelatase
GVHD: graft *versus* host disease
HbA: adult hemoglobin
HbF: fetal hemoglobin
HbS: hemoglobin S
HSCs: hematopoietic stem cells
HSCT: HSC transplantation
HU: Hydroxyurea
iPSCs: induced Pluripotent Stem Cells
PEG: polyethylene glycol
PRT: protein replacement therapy
PTD: protein transduction domain
PTs: proteins therapeutics
R: arginine
RBC: red blood cell
SCD: sickle cell disease
α: alpha
β: beta
γ: gamma
δ: delta

## References

[1] Tsiftsoglou A.S., Vizirianakis I.S., Strouboulis J. (2009). Erythropoiesis: Model systems, molecular regulators, and developmental program. IUBMB Life.

[2] Jelkmann W. (2011). Regulation of erythropoietin production. J. Physiol..

[3] Schechter A.N. (2008). Hemoglobin research and the origins of molecular medicine. Blood.

[4] Higgs D.R., Engel J.D., Stamatoyannopoulos G. (2012). Thalassaemia. Lancet.

[5] An X., Mohandas N. (2008). Disorders of red cell membrane. Br. J. Haematol..

[6] Foller M., Huber S.M., Lang F. (2008). Erythrocyte programmed cell death. IUBMB Life.

[7] Voon H.P., Vadolas J. (2008). Controlling alpha-globin: A review of alpha-globin expression and its impact on beta-thalassemia. Haematologica.

[8] Komar A.A., Kommer A., Krasheninnikov I.A., Spirin A.S. (1997). Cotranslational folding of globin. J. Biol. Chem..

[9] Komar A.A., Kommer A., Krasheninnikov I.A., Spirin A.S. (1993). Cotranslational heme binding to nascent globin chains. FEBS Lett..

[10] Mollan T.L., Yu X., Weiss M.J., Olson J.S. (2010). The role of alpha-hemoglobin stabilizing protein in redox chemistry, denaturation, and hemoglobin assembly. Antioxid Redox Signal..

[11] Elder G.H. (1993). Molecular genetics of disorders of haem biosynthesis. J. Clin. Pathol..

[12] Tanno T., Miller J.L. (2010). Iron loading and overloading due to ineffective erythropoiesis. Adv. Hematol..

[13] Wang S.A., Hasserjian R.P. (2012). Erythroid proliferations in myeloid neoplasms. Hum. Pathol..

[14] Kalb R., Neveling K., Nanda I., Schindler D., Hoehn H. (2006). Fanconi anemia: Causes and consequences of genetic instability. Genome Dyn..

[15] Xu X., Qu J., Suzuki K., Li M., Zhang W., Liu G.H., Izpisua Belmonte J.C. (2012). Reprogramming based gene therapy for inherited red blood cell disorders. Cell. Res..

[16] Storb R.F., Lucarelli G., McSweeney P.A., Childs R.W. (2003). Hematopoietic cell transplantation for benign hematological disorders and solid tumors. Hematology Am. Soc. Hematol. Educ. Program..

[17] Snyder E.L., Dowdy S.F. (2005). Recent advances in the use of protein transduction domains for the delivery of peptides, proteins and nucleic acids *in vivo*. Expert Opin. Drug Deliv..

[18] Papadopoulou L.C., Tsiftsoglou A.S. (2011). Transduction of human recombinant proteins into mitochondria as a protein therapeutic approach for mitochondrial disorders. Pharm. Res..

[19] Suzuki Y. (2012). Exploring transduction mechanisms of protein transduction domains (ptds) in living cells utilizing single-quantum dot tracking (sqt) technology. Sensors (Basel).

[20] Fleming M.D. (2011). Congenital sideroblastic anemias: Iron and heme lost in mitochondrial translation. Hematology Am. Soc. Hematol. Educ. Program..

[21] Ajioka R.S., Phillips J.D., Kushner J.P. (2006). Biosynthesis of heme in mammals. Biochim. Biophys. Acta.

[22] Harigae H., Furuyama K. (2010). Hereditary sideroblastic anemia: Pathophysiology and gene mutations. Int.J. Hematol..

[23] Bishop D.F., Tchaikovskii V., Hoffbrand A.V., Fraser M.E., Margolis S. (2012). X-linked sideroblastic anemia due to carboxyl-terminal alas2 mutations that cause loss of binding to the beta-subunit of succinyl-coa synthetase (sucla2). J. Biol. Chem..

[24] Tsiftsoglou A.S., Tsamadou A.I., Papadopoulou L.C. (2006). Heme as key regulator of major mammalian cellular functions: Molecular, cellular, and pharmacological aspects. Pharmacol. Ther..

[25] Fontenay M., Cathelin S., Amiot M., Gyan E., Solary E. (2006). Mitochondria in hematopoiesis and hematological diseases. Oncogene.

[26] Cuijpers M.L., van Spronsen D.J., Muus P., Hamel B.C., Swinkels D.W. (2011). Need for early recognition and therapeutic guidelines of congenital sideroblastic anaemia. Int. J. Hematol..

[27] Baumann Kreuziger L.M., Wolanskyj A.P., Hanson C.A., Steensma D.P. (2011). Lack of efficacy of pyridoxine (vitamin b6) treatment in acquired idiopathic sideroblastic anaemia, including refractory anaemia with ring sideroblasts. Eur. J. Haematol..

[28] Guernsey D.L., Jiang H., Campagna D.R., Evans S.C., Ferguson M., Kellogg M.D., Lachance M., Matsuoka M., Nightingale M., Rideout A., Saint-Amant L., Schmidt P.J., Orr A., Bottomley S.S., Fleming M.D., Ludman M., Dyack S., Fernandez C.V., Samuels M.E. (2009). Mutations in mitochondrial carrier family gene slc25a38 cause nonsyndromic autosomal recessive congenital sideroblastic anemia. Nat. Genet..

[29] Khan A.A., Quigley J.G. (2011). Control of intracellular heme levels: Heme transporters and heme oxygenases. Biochim. Biophys. Acta.

[30] Zutz A., Gompf S., Schagger H., Tampe R. (2009). Mitochondrial abc proteins in health and disease. Biochim. Biophys. Acta.

[31] Allikmets R., Raskind W.H., Hutchinson A., Schueck N.D., Dean M., Koeller D.M. (1999). Mutation of a putative mitochondrial iron transporter gene (abc7) in x-linked sideroblastic anemia and ataxia (xlsa/a). Hum. Mol. Genet..

[32] Ye H., Jeong S.Y., Ghosh M.C., Kovtunovych G., Silvestri L., Ortillo D., Uchida N., Tisdale J., Camaschella C., Rouault T.A. (2010). Glutaredoxin 5 deficiency causes sideroblastic anemia by specifically impairing heme biosynthesis and depleting cytosolic iron in human erythroblasts. J. Clin. Invest..

[33] Pearson H.A., Lobel J.S., Kocoshis S.A., Naiman J.L., Windmiller J., Lammi A.T., Hoffman R., Marsh J.C. (1979). A new syndrome of refractory sideroblastic anemia with vacuolization of marrow precursors and exocrine pancreatic dysfunction. J. Pediatr..

[34] Rawles J.M., Weller R.O. (1974). Familial association of metabolic myopathy, lactic acidosis and sideroblastic anemia. Am. J. Med..

[35] Porter F.S., Rogers L.E., Sidbury J.B. (1969). Thiamine-responsive megaloblastic anemia. J. Pediatr..

[36] Siegesmund M., van Tuyll van Serooskerken A.M., Poblete-Gutierrez P., Frank J. (2010). The acute hepatic porphyrias: Current status and future challenges. Best Pract. Res. Clin. Gastroenterol..

[37] Hift R.J., Thunell S., Brun A. (2011). Drugs in porphyria: From observation to a modern algorithm-based system for the prediction of porphyrogenicity. Pharmacol.Ther..

[38] Cappellini M.D., Brancaleoni V., Graziadei G., Tavazzi D., Di Pierro E. (2010). Porphyrias at a glance: Diagnosis and treatment. Intern. Emerg. Med..

[39] Hunter G.A., Ferreira G.C. (2011). Molecular enzymology of 5-aminolevulinate synthase, the gatekeeper of heme biosynthesis. Biochim. Biophys. Acta.

[40] Schmitt C., Ducamp S., Gouya L., Deybach J.C., Puy H. (2010). [inheritance in erythropoietic protoporphyria]. Pathol. Biol. (Paris).

[41] Phillips J.D., Steensma D.P., Pulsipher M.A., Spangrude G.J., Kushner J.P. (2007). Congenital erythropoietic porphyria due to a mutation in gata1: The first trans-acting mutation causative for a human porphyria. Blood.

[42] May B.K., Dogra S.C., Sadlon T.J., Bhasker C.R., Cox T.C., Bottomley S.S. (1995). Molecular regulation of heme biosynthesis in higher vertebrates. Prog. Nucleic. Acid. Res. Mol. Biol..

[43] Simon N.G., Herkes G.K. (2011). The neurologic manifestations of the acute porphyrias. J. Clin. Neurosci..

[44] Perutz M.F., Lehmann H. (1968). Molecular pathology of human haemoglobin. Nature.

[45] Angastiniotis M., Modell B. (1998). Global epidemiology of hemoglobin disorders. Ann. N Y Acad. Sci..

[46] Birgens H., Ljung R. (2007). The thalassaemia syndromes. Scand. J. Clin. Lab. Invest..

[47] Weatherall D.J. (2010). Thalassemia as a global health problem: Recent progress toward its control in the developing countries. Ann. N Y Acad. Sci..

[48] Weatherall D.J. (2008). Genetic variation and susceptibility to infection: The red cell and malaria. Br. J. Haematol..

[49] Giardine B., van Baal S., Kaimakis P., Riemer C., Miller W., Samara M., Kollia P., Anagnou N.P., Chui D.H., Wajcman H., Hardison R.C., Patrinos G.P. (2007). Hbvar database of human hemoglobin variants and thalassemia mutations: 2007 update. Hum. Mutat..

[50] Urbinati F., Madigan C., Malik P. (2006). Pathophysiology and therapy for haemoglobinopathies. Part ii: Thalassaemias. Expert Rev. Mol. Med..

[51] Muncie H.L., Campbell J. (2009). Alpha and beta thalassemia. Am. Fam. Physician.

[52] Harteveld C.L., Higgs D.R. (2010). Alpha-thalassaemia. Orphanet. J. Rare Dis..

[53] Bourantas K., Economou G., Georgiou J. (1997). Administration of high doses of recombinant human erythropoietin to patients with beta-thalassemia intermedia: A preliminary trial. Eur. J. Haematol..

[54] Gambari R. (2012). Alternative options for DNA-based experimental therapy of beta-thalassemia. Expert Opin. Biol. Ther..

[55] Stamatoyannopoulos G. (2005). Control of globin gene expression during development and erythroid differentiation. Exp. Hematol..

[56] Hankins J., Aygun B. (2009). Pharmacotherapy in sickle cell disease--state of the art and future prospects. Br. J. Haematol.

[57] Ataga K.I. (2009). Novel therapies in sickle cell disease. Hematology Am. Soc. Hematol. Educ. Program..

[58] Elborai Y., Uwumugambi A., Lehmann L. (2012). Hematopoietic stem cell transplantation for thalassemia. Immunotherapy.

[59] Mogul M.J. (2000). Unrelated cord blood transplantation vs matched unrelated donor bone marrow transplantation: The risks and benefits of each choice. Bone Marrow Transplant..

[60] Quek L., Thein S.L. (2007). Molecular therapies in beta-thalassaemia. Br. J. Haematol..

[61] Cavazzana-Calvo M., Payen E., Negre O., Wang G., Hehir K., Fusil F., Down J., Denaro M., Brady T., Westerman K., Cavallesco R., Gillet-Legrand B., Caccavelli L., Sgarra R., Maouche-Chretien L., Bernaudin F., Girot R., Dorazio R., Mulder G.J., Polack A., Bank A., Soulier J., Larghero J., Kabbara N., Dalle B., Gourmel B., Socie G., Chretien S., Cartier N., Aubourg P., Fischer A., Cornetta K., Galacteros F., Beuzard Y., Gluckman E., Bushman F., Hacein-Bey-Abina S., Leboulch P. (2010). Transfusion independence and hmga2 activation after gene therapy of human beta-thalassaemia. Nature.

[62] Sarakul O., Vattanaviboon P., Wilairat P., Fucharoen S., Abe Y., Muta K. (2008). Inhibition of alpha-globin gene expression by rnai. Biochem. Biophys. Res. Commun..

[63] Vadolas J., Wardan H., Orford M., Williamson R., Ioannou P.A. (2004). Cellular genomic reporter assays for screening and evaluation of inducers of fetal hemoglobin. Hum. Mol. Genet..

[64] Rees D.C., Williams T.N., Gladwin M.T. (2010). Sickle-cell disease. Lancet.

[65] Boosalis M.S., Castaneda S.A., Trudel M., Mabaera R., White G.L., Lowrey C.H., Emery D.W., Mpollo M.S., Shen L., Wargin W.A., Bohacek R., Faller D.V., Perrine S.P. (2011). Novel therapeutic candidates, identified by molecular modeling, induce gamma-globin gene expression in vivo. Blood Cells Mol. Dis..

[66] Samakoglu S., Lisowski L., Budak-Alpdogan T., Usachenko Y., Acuto S., Di Marzo R., Maggio A., Zhu P., Tisdale J.F., Riviere I., Sadelain M. (2006). A genetic strategy to treat sickle cell anemia by coregulating globin transgene expression and rna interference. Nat. Biotechnol..

[67] Hanna J., Wernig M., Markoulaki S., Sun C.W., Meissner A., Cassady J.P., Beard C., Brambrink T., Wu L.C., Townes T.M., Jaenisch R. (2007). Treatment of sickle cell anemia mouse model with ips cells generated from autologous skin. Science.

[68] Douay L. (2012). In vitro generation of red blood cells for transfusion: A model for regenerative medicine. Regen. Med..

[69] Migliaccio A.R., Masselli E., Varricchio L., Whitsett C. (2012). Ex-vivo expansion of red blood cells: How real for transfusion in humans?. Blood Rev..

[70] Stephens D.J., Pepperkok R. (2001). The many ways to cross the plasma membrane. Proc. Natl. Acad. Sci. USA.

[71] Fawell S., Seery J., Daikh Y., Moore C., Chen L.L., Pepinsky B., Barsoum J. (1994). Tat-mediated delivery of heterologous proteins into cells. Proc. Natl. Acad. Sci. USA.

[72] Derossi D., Joliot A.H., Chassaing G., Prochiantz A. (1994). The third helix of the antennapedia homeodomain translocates through biological membranes. J. Biol. Chem..

[73] Frankel A.D., Pabo C.O. (1988). Cellular uptake of the tat protein from human immunodeficiency virus. Cell.

[74] Brasseur R., Divita G. (2010). Happy birthday cell penetrating peptides: Already 20years. Biochim. Biophys. Acta.

[75] Vives E. (2005). Present and future of cell-penetrating peptide mediated delivery systems: "Is the trojan horse too wild to go only to troy?". J. Control. Release.

[76] Snyder E.L., Dowdy S.F. (2004). Cell penetrating peptides in drug delivery. Pharm Res..

[77] Eguchi A., Dowdy S.F. (2009). Sirna delivery using peptide transduction domains. Trends Pharmacol. Sci..

[78] El Andaloussi S.A., Hammond S.M., Mager I., Wood M.J. (2012). Use of cell-penetrating-peptides in oligonucleotide splice switching therapy. Curr. Gene Ther..

[79] Gautam A., Singh H., Tyagi A., Chaudhary K., Kumar R., Kapoor P., Raghava G.P.  (2012). Cppsite: A curated database of cell penetrating peptides. Database (Oxford).

[80] Milletti F. (2012). Cell-penetrating peptides: Classes, origin, and current landscape. Drug Discov. Today.

[81] Schwarze S.R., Ho A., Vocero-Akbani A., Dowdy S.F. (1999). In vivo protein transduction: Delivery of a biologically active protein into the mouse. Science.

[82] Elliott G., O'Hare P. (1997). Intercellular trafficking and protein delivery by a herpesvirus structural protein. Cell.

[83] Futaki S., Suzuki T., Ohashi W., Yagami T., Tanaka S., Ueda K., Sugiura Y.  (2001). Arginine-rich peptides. An abundant source of membrane-permeable peptides having potential as carriers for intracellular protein delivery. J. Biol. Chem..

[84] Wender P.A., Cooley C.B., Geihe E.I. (2012). Beyond cell penetrating peptides: Designed molecular transporters. Drug Discov. Today Technol..

[85] Lindgren M., Gallet X., Soomets U., Hallbrink M., Brakenhielm E., Pooga M., Brasseur R., Langel U. (2000). Translocation properties of novel cell penetrating transportan and penetratin analogues. Bioconjug. Chem..

[86] Soomets U., Lindgren M., Gallet X., Hallbrink M., Elmquist A., Balaspiri L., Zorko M., Pooga M., Brasseur R., Langel U. (2000). Deletion analogues of transportan. Biochim. Biophys. Acta.

[87] Elmquist A., Lindgren M., Bartfai T., Langel U. (2001). Ve-cadherin-derived cell-penetrating peptide, pvec, with carrier function. Exp. Cell. Res..

[88] Gros E., Deshayes S., Morris M.C., Aldrian-Herrada G., Depollier J., Heitz F., Divita G. (2006). A non-covalent peptide-based strategy for protein and peptide nucleic acid transduction. Biochim. Biophys. Acta.

[89] Rhee M., Davis P. (2006). Mechanism of uptake of c105y, a novel cell-penetrating peptide. J. Biol. Chem..

[90] Crombez L., Aldrian-Herrada G., Konate K., Nguyen Q.N., McMaster G.K., Brasseur R., Heitz F., Divita G. (2009). A new potent secondary amphipathic cell-penetrating peptide for sirna delivery into mammalian cells. Mol. Ther..

[91] Kalafut D., Anderson T.N., Chmielewski J. (2012). Mitochondrial targeting of a cationic amphiphilic polyproline helix. Bioorg Med. Chem. Lett..

[92] Green I., Christison R., Voyce C.J., Bundell K.R., Lindsay M.A. (2003). Protein transduction domains: Are they delivering?. Trends Pharmacol. Sci..

[93] Fischer R., Fotin-Mleczek M., Hufnagel H., Brock R. (2005). Break on through to the other side-biophysics and cell biology shed light on cell-penetrating peptides. Chembiochem.

[94] Mueller J., Kretzschmar I., Volkmer R., Boisguerin P. (2008). Comparison of cellular uptake using 22 cpps in 4 different cell lines. Bioconjug. Chem..

[95] Koren E., Torchilin V.P. (2012). Cell-penetrating peptides: Breaking through to the other side. Trends Mol. Med..

[96] Jones A.T., Sayers E.J. (2012). Cell entry of cell penetrating peptides: Tales of tails wagging dogs. J. Control. Release.

[97] Sung M., Poon G.M., Gariepy J. (2006). The importance of valency in enhancing the import and cell routing potential of protein transduction domain-containing molecules. Biochim Biophys Acta.

[98] Vocero-Akbani A., Lissy N.A., Dowdy S.F. (2000). Transduction of full-length tat fusion proteins directly into mammalian cells: Analysis of t cell receptor activation-induced cell death. Methods Enzymol..

[99] Foltopoulou P.F., Tsiftsoglou A.S., Bonovolias I.D., Ingendoh A.T., Papadopoulou L.C. (2010). Intracellular delivery of full length recombinant human mitochondrial l-sco2 protein into the mitochondria of permanent cell lines and sco2 deficient patient's primary cells. Biochim. Biophys. Acta.

[100] Flinterman M., Farzaneh F., Habib N., Malik F., Gaken J., Tavassoli M. (2009). Delivery of therapeutic proteins as secretable tat fusion products. Mol. Ther..

[101] Chen X., Bai Y., Zaro J.L., Shen W.C. (2010). Design of an *in vivo* cleavable disulfide linker in recombinant fusion proteins. Biotechniques.

[102] Zaro J.L., Fei L., Shen W.C. (2012). Recombinant peptide constructs for targeted cell penetrating peptide-mediated delivery. J. Control. Release.

[103] Sawant R.M., Hurley J.P., Salmaso S., Kale A., Tolcheva E., Levchenko T.S., Torchilin V.P. (2006). "Smart" drug delivery systems: Double-targeted ph-responsive pharmaceutical nanocarriers. Bioconjug. Chem..

[104] Sawant R.R., Jhaveri A.M., Torchilin V.P. (2012). Immunomicelles for advancing personalized therapy. Adv. Drug Deliv. Rev..

[105] Aubry S., Burlina F., Dupont E., Delaroche D., Joliot A., Lavielle S., Chassaing G., Sagan S. (2009). Cell-surface thiols affect cell entry of disulfide-conjugated peptides. FASEB J..

[106] Kale A.A., Torchilin V.P. (2007). "Smart" drug carriers: Pegylated tatp-modified ph-sensitive liposomes. J. Liposome Res..

[107] Khafagy el S., Morishita M. (2012). Oral biodrug delivery using cell-penetrating peptide. Adv. Drug Deliv. Rev..

[108] Liu B.R., Li J.F., Lu S.W., Leel H.J., Huang Y.W., Shannon K.B., Aronstam R.S. (2010). Cellular internalization of quantum dots noncovalently conjugated with arginine-rich cell-penetrating peptides. J. Nanosci. Nanotechnol..

[109] Hu J.W., Liu B.R., Wu C.Y., Lu S.W., Lee H.J. (2009). Protein transport in human cells mediated by covalently and noncovalently conjugated arginine-rich intracellular delivery peptides. Peptides.

[110] Jarver P., Mager I., Langel U. (2010). In vivo biodistribution and efficacy of peptide mediated delivery. Trends Pharmacol. Sci..

[111] Snyder E.L., Saenz C.C., Denicourt C., Meade B.R., Cui X.S., Kaplan I.M., Dowdy S.F. (2005). Enhanced targeting and killing of tumor cells expressing the cxc chemokine receptor 4 by transducible anticancer peptides. Cancer Res..

[112] Heffernan C., Sumer H., Guillemin G.J., Manuelpillai U., Verma P.J. (2012). Design and screening of a glial cell-specific, cell penetrating peptide for therapeutic applications in multiple sclerosis. PLoS One.

[113] Verdurmen W.P., Bovee-Geurts P.H., Wadhwani P., Ulrich A.S., Hallbrink M., van Kuppevelt T.H., Brock R. (2011). Preferential uptake of l-*versus* d-amino acid cell-penetrating peptides in a cell type-dependent manner. Chem. Biol..

[114] Wang H., Zhang S., Liao Z., Wang C., Liu Y., Feng S., Jiang X., Chang J. (2010). Peglated magnetic polymeric liposome anchored with tat for delivery of drugs across the blood-spinal cord barrier. Biomaterials.

[115] Weber C.A., Mehta P.J., Ardito M., Moise L., Martin B., De Groot A.S. (2009). T cell epitope: Friend or foe? Immunogenicity of biologics in context. Adv. Drug Deliv. Rev..

[116] Bryson C.J., Jones T.D., Baker M.P. (2010). Prediction of immunogenicity of therapeutic proteins: Validity of computational tools. BioDrugs.

[117] Schellekens H. (2002). Bioequivalence and the immunogenicity of biopharmaceuticals. Nat. Rev. Drug Discov..

[118] Sarko D., Beijer B., Boy R.G., Nothelfer E.M., Leotta K., Eisenhut M., Altmann A., Haberkorn U., Mier W. (2010). The pharmacokinetics of cell-penetrating peptides. Mol. Pharm..

[119] D'Souza G.G., Weissig V. (2009). Subcellular targeting: A new frontier for drug-loaded pharmaceutical nanocarriers and the concept of the magic bullet. Expert Opin. Drug Deliv..

[120] Mossalam M., Dixon A.S., Lim C.S. (2010). Controlling subcellular delivery to optimize therapeutic effect. Ther. Deliv..

[121] Davis J.R., Kakar M., Lim C.S. (2007). Controlling protein compartmentalization to overcome disease. Pharm. Res..

[122] Rapoport M., Salman L., Sabag O., Patel M.S., Lorberboum-Galski H. (2011). Successful tat-mediated enzyme replacement therapy in a mouse model of mitochondrial e3 deficiency. J. Mol. Med..

[123] Macewan S.R., Chilkoti A. (2012). Harnessing the power of cell-penetrating peptides: Activatable carriers for targeting systemic delivery of cancer therapeutics and imaging agents. Wiley Interdiscip. Rev. Nanomed. Nanobiotechnol..

[124] Johnson R.M., Harrison S.D., Maclean D. (2010). Therapeutic applications of cell-penetrating peptides. Methods Mol. Biol..

[125] van den Berg A., Dowdy S.F. (2011). Protein transduction domain delivery of therapeutic macromolecules. Curr. Opin. Biotechnol..

[126] Rothbard J.B., Garlington S., Lin Q., Kirschberg T., Kreider E., McGrane P.L., Wender P.A., Khavari P.A. (2000). Conjugation of arginine oligomers to cyclosporin a facilitates topical delivery and inhibition of inflammation. Nat. Med..

[127] Lopes L.B., Furnish E.J., Komalavilas P., Flynn C.R., Ashby P., Hansen A., Ly D.P., Yang G.P., Longaker M.T., Panitch A., Brophy C.M. (2009). Cell permeant peptide analogues of the small heat shock protein, hsp20, reduce tgf-beta1-induced ctgf expression in keloid fibroblasts. J. Invest. Dermatol..

[128] Lebleu B., Moulton H.M., Abes R., Ivanova G.D., Abes S., Stein D.A., Iversen P.L., Arzumanov A.A., Gait M.J. (2008). Cell penetrating peptide conjugates of steric block oligonucleotides. Adv. Drug Deliv. Rev..

[129] Ensoli B., Bellino S., Tripiciano A., Longo O., Francavilla V., Marcotullio S., Cafaro A., Picconi O., Paniccia G., Scoglio A., Arancio A., Ariola C., Ruiz Alvarez M.J., Campagna M., Scaramuzzi D., Iori C., Esposito R., Mussini C., Ghinelli F., Sighinolfi L., Palamara G., Latini A., Angarano G., Ladisa N., Soscia F., Mercurio V.S., Lazzarin A., Tambussi G., Visintini R., Mazzotta F., Di Pietro M., Galli M., Rusconi S., Carosi G., Torti C., Di Perri G., Bonora S., Ensoli F., Garaci E. (2010). Therapeutic immunization with Hiv-1 tat reduces immune activation and loss of regulatory t-cells and improves immune function in subjects on haart. PLoS One.

[130] Eguchi A., Dowdy S.F. (2010). Efficient sirna delivery by novel ptd-drbd fusion proteins. Cell. Cycle.

[131] Palm-Apergi C., Eguchi A., Dowdy S.F. (2011). Ptd-drbd sirna delivery. Methods Mol. Biol..

[132] Reinecke K., Eminel S., Dierck F., Roessner W., Kersting S., Chromik A.M., Gavrilova O., Laukevicience A., Leuschner I., Waetzig V., Rosenstiel P., Herdegen T., Sina C. (2012). The jnk inhibitor xg-102 protects against tnbs-induced colitis. PLoS One.

[133] Meyer-Losic F., Nicolazzi C., Quinonero J., Ribes F., Michel M., Dubois V., de Coupade C., Boukaissi M., Chene A.S., Tranchant I., Arranz V., Zoubaa I., Fruchart J.S., Ravel D., Kearsey J. (2008). Dts-108, a novel peptidic prodrug of sn38: In vivo efficacy and toxicokinetic studies. Clin. Cancer Res..

[134] Drakopoulou E., Papanikolaou E., Anagnou N.P. (2011). The ongoing challenge of hematopoietic stem cell-based gene therapy for beta-thalassemia. Stem Cells Int..

